# A Large Sublingual Dermoid Cyst Causing Dysphagia and Dysphonia: A Case Review Study

**DOI:** 10.1155/2023/7776558

**Published:** 2023-07-28

**Authors:** Mohsen Barzegar, Yasamin Akhavan-Tafti, Hamid Reza Soltani, Mohammad Goodarzi

**Affiliations:** ^1^Department of Oral and Maxillofacial Surgery, Shahid Sadoughi University of Medical Sciences and Health Services, Yazd, Iran; ^2^Department of Oral and Maxillofacial Radiology, Shahid Sadoughi University of Medical Sciences and Health Services, Yazd, Iran; ^3^Department of General Surgery, Shahid Sadoughi University of Medical Sciences and Health Services, Yazd, Iran

## Abstract

A seventeen-year-old girl was referred to the emergency department with dysphagia and dyspnea due to large swelling in the floor of the mouth after 20 days of evaluation. Magnetic resonance imaging shows a well-defined sublingual mass measuring 70 mm × 74 mm × 46 mm, causing severe oral and oropharyngeal space narrowing. The surgical excision of the lesion was performed through an intraoral approach under general anesthesia. Moreover, the pathologist reported a dermoid cyst. A dermoid cyst rapidly enlarging can lead to a life-threatening condition, particularly if they grow near main upper airway structures, so their resection in golden time has an especially clinical importance.

## 1. Introduction

The dermoid cyst is a rare benign cutaneous neoplasm tumor arising from ectoderm and mesoderm [1] with equal incidence in both sexes [2]. About 80% arise in the ovaries and sacral regions [3, 4], but dermoid cysts in the floor of the mouth are 1–1.6% of all of them [5, 6]. Cysts have three types of locations: (1) sublingual; (2) submandibular; and (3) submental [7, 8]. Clinically, mostly, it is an asymptomatic tumor that grows slowly but, in the case of enlarging, can cause some difficulties like dysphagia, dysphonia, and dyspnea [9]. Some approaches for its diagnosis are considered, like ultrasonography, computed tomography (CT) scan, and magnetic resonance imaging (MRI) [10]. The oral approach for surgical removal of the lesion is the treatment of choice [11–13]. This report presents a rare case of a huge epidermoid cyst in the floor of the mouth, causing dysphagia.

## 2. Case Presentation

A seventeen-year-old girl was referred to the emergency department with dysphagia and dyspnea due to large swelling in the floor of the mouth after 20 days of evaluation. The patient found dysphonia and swallowing difficulties during the last three months. His medical history and family history were unremarkable. Extraoral examination presented a palpable swelling in the midline of the neck that caused the tongue to buckle in the oral cavity. The lesion was slightly movable and tender on palpation. Aspiration was done, and about 50 cc of keratin-containing liquid were aspirated. There was no palpable lateral cervical lymphadenopathy or fever (temperature: 36.5°C).

**Figure 1:**
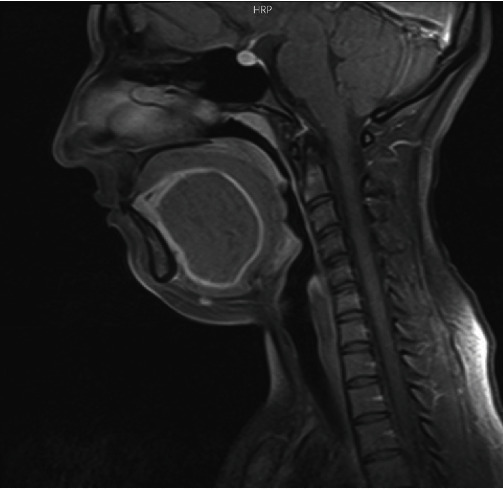
MRI showing a well-defined mass in the sublingual area.

Magnetic resonance imaging shows a well-defined sublingual mass measuring 70 mm × 74 mm × 46 mm, causing severe narrowing in the oral and oropharyngeal spaces (Figure 1).

**Figure 2:**
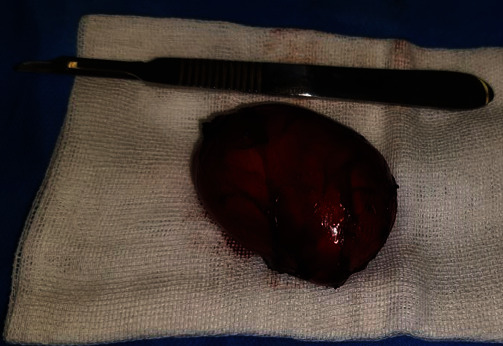
Complete mass excision.

**Figure 3:**
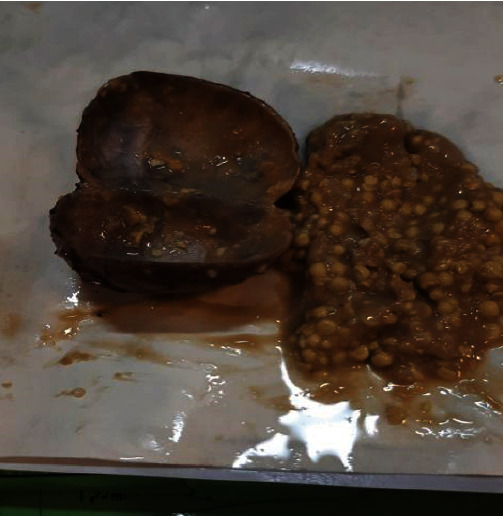
The encapsulated mass contained a keratin-like yellow material.

**Figure 4:**
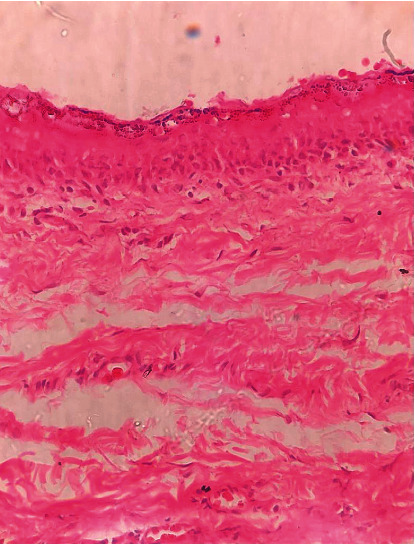
Pathologic view of the lesion in favor of dermoid cyst.

Surgical excision of the lesion was performed (Figure 2) through an intraoral approach under general anesthesia. After injection of lidocaine 2% with epinephrine 1/200,000 for hemostasis, the incision was made on the floor of the mouth, followed by blunt dissection. The cyst was dissected entirely from the muscle and removed. The wound was closed primarily, and to avoid hematoma, a blaster suture of the floor of the mouth was applied to the neck skin, and the tongue retraction suture was fixed. Macroscopically, the lesion appeared encapsulated and contained a keratin-like yellow material (Figure 3). The pathologic diagnosis was an epidermoid cyst (Figure 4). The patient was discharged after four days without complications. There is no recurrence after six months of follow-up.

## 3. Discussion

Dermoid cysts include keratinized stratified squamous epithelial linings incorporating adnexal structures like hair follicles, sebaceous glands, and eccrine glands [14]. These adnexal structures can differentiate between a dermoid and an epidermoid cyst [15]. They are benign tumors with the slow-growing origin of dermoid cysts and are both congenital and acquired manners [16]. Although this theory is still unknown, there are two theories about its origin. The most accepted supposition is a congenital origin, and defective fusion of the mandibular and branchial arches occurs in the third and fourth weeks of development. As a result, entrap ectodermic tissue in the midline [17, 18].

Furthermore, the second accepted supposition resulting from trauma or iatrogenic implantation of epithelial cell are in surrounding tissues [19], especially in utero [20, 21]. One of the first people that define and describe the dermoid cyst of the floor of the mouth was Jurdain's Traite in 1778 [22]. Usually, the development of a dermoid cyst will be arch with patients not becoming aware till enough enlargement interferes with speaking, swallowing, or eating [23]. These disabling conditions were seen in our case also. Dermoid cysts were located below the geniohyoid, and a submental swelling with a double chin appearance may occur [23]. The clinical and radiographic findings of a dermoid cyst may be doubtful. CT scan, MRI, ultrasound, and FNA can be accomplished, estimating size and location and relationship to the adjacent structure of the cyst with CT scan and MRI exactly. However, these are not definitive preoperative diagnoses. The first line of the diagnostic procedure in a dermoid cyst on the floor of the mouth should be fine needle aspiration cytology (FNAC) [24]. MRI is the gold standard imaging modality for diagnosing cystic masses [25, 26]. In the diagnostic image, you can see intracystic floating corpuscles that contain lipids (i.e., the sack of marble sign). These are pathognomonic for dermoid cysts and usually hypodense on CT, hyper/hypo intense on T1-weighted MRI, and hypointense on T2-weighted MRI, although cystic hygroma, branchial cleft cyst, sublingual ranula, and benign and malignant tumors of the mouth are all considered as radiological differential diagnosis of dermoid cyst [27, 28]. Because of these marbles, a percutaneous fine needle aspiration may not be recommended. During the time these marbles can be calcified, selecting a surgical approach with the location of the cyst. The most recommended intraoral and extraoral approaches for sublingual cysts are usually recommended for submandibular and submental dermoid cysts [29, 30]. A list of similar cases with their detailed clinical pathways is shown in Table 1.

On the other hand, blind aspiration without an adequate imaging study may lead to massive bleeding because hemangiomas are considered differential diagnoses. The standard gold treatment for dermoid cysts is surgical enucleation, which makes it easier due to the capsule [3]. After surgical excision, recurrence is uncommon [29, 30]. Probably in the future may become infected or, in rare cases, find malignancy occurrence, so it is cause for exclusion (removal) [23] as was done in this current case.

## 4. Conclusion

Although dermoid cysts are benign tumors, noticing another differential diagnosis like hemangioma is important because blind aspiration is a common diagnostic approach without an adequate imaging study that should be considered. On the other hand, their rapid enlarging can lead to life-threatening conditions, particularly if they grow near main upper airway structures, so their resection in golden time has an especially clinical importance.

## Figures and Tables

**Table 1:** Similar reported cases of dermoid cyst with their clinical pathway from 2009 until now.

	Author	Year	Size (mm)	Location	Chief complaint	Diagnosing tool	Treatment	Follow up
1	Ulku and Yucel [31]	2015	20 × 30	Epiglottis	Muffled voice and dysphagia	Endoscopic examination and MRI	Surgical excision	Normal status (6 months)
2	Dwivedi et al. [32]	2019	40 × 50	The floor of the mouth and upper neck	Painless swelling	Ultrasonography and MRI	Cervical incision	Normal status (6 months)
3	Ohta et al. [18]	2012	60 × 50	Sublingual	Difficultly chewing and swallowing	MRI and aspiration	Intraoral excision	Normal status (6 months)
4	Aydin et al. [33]	2016	40 × 40	Sublingual	Mass palpation	MRI	Intraoral excision	Normal status (6 months)
5	Jadwani et al. [3]	2009	40 × 20	Right side of floor of the mouth	Asymptomatic	FNAC and aspiration	Surgical (intraoral excision)	Normal status during 2 years
6	Giarraputo et al. [34]	2018	20 × 15 × 15	Submandibular	Painless mass	Ultrasonography and MRI and CT	Surgical (submandibular incision)	Normal status (6 months)
7	Berbel et al. [35]	2016	60 × 50	Sublingual	Lip incompetency and salivary incontinence	CT	Surgical (sublingual incision)	Normal status during 12 months
8	Patel et al. [9]	2022	4.6 × 8.8	Submental (floor of the mouth)	Dysphagia and dysphonia	CT and aspiration	Surgical (transoral approach)	Not reported
